# Supplementary material for: High Myopia and Its Associated Factors in JPHC-NEXT Eye Study: A Cross-Sectional Observational Study
Source: J Clin Med. 2019 Oct 25;8(11):1788. doi: 10.3390/jcm8111788 (PMC6912595; doi:10.3390/jcm8111788)
Supplement: Supplementary file 1 [file jcm-08-01788-s001.pdf]

Supplementary Materials

Table S1. The association between alcohol, tobacco and high myopia.

Variables		Non High Myopia	High Myopia	*p* value	OR	95% CI	*p* value
		History/no History	History/no History				
Alcohol							
	men	481/187	12/4	1.000	1.17	(0.371–3.662)	0.792
	women	214/722	12/45	0.871	0.90	(0.467–1.732)	0.752
Tobacco							
	men	1134/339	38/19	0.079	0.60	(0.340–1.051)	0.074
	women	299/1750	20/121	1.000	0.97	(0.593–1.577)	0.894

Table S2. Logistic regression analysis to identify factors associated with high myopia.

		High Myopia (number)	Non High Myopia (number)	OR 1	95% CI	*p* value	OR 2	95% CI	*p* value
Men									
Age	≤58	43	552	1			1		
	59–65	21	574	0.47	0.275–0.802		0.44	0.221–0.886	
	66–70	13	523	0.32	0.170–0.600		0.43	0.197–0.930	
	≥71	15	686	0.28	0.154–0.511		0.48	0.219–1.059	
	p for trend					<0.001			0.049
HT	≤160	16	589	1			1		
	161–164	15	537	0.92	0.447–1.894		0.95	0.388–2.318	
	165–168	24	563	1.20	0.613–2.335		1.42	0.620–3.271	
	≥169	37	646	1.27	0.659–2.443		1.65	0.722–3.784	
	p for trend					0.343			0.131
BMI	≤21.6	20	577	1			1		
	21.7–23.5	26	571	1.28	0.703–2.326		1.41	0.660–3.020	
	23.6–25.6	17	583	0.84	0.435–1.630		0.96	0.423–2.188	
	≥25.7	28	586	1.24	0.687–2.239		1.07	0.479–2.368	
	p for trend					0.750			0.836
SBP	≤116	22	572	1			1		
	117–127	19	560	1.04	0.551–1.948		0.61	0.272–1.364	
	128–138	28	567	1.64	0.912–2.928		0.98	0.481–1.978	
	≥139	22	619	1.26	0.680–2.343		0.83	0.396–1.741	
	p for trend					0.304			0.912
TG	≤73	7	508	1			1		
	74–105	20	521	2.77	1.157–6.614		2.93	1.126–7.616	
	106–149	22	505	3.18	1.343–7.529		3.10	1.183–8.103	
	≥150	22	522	2.76	1.162–6.541		2.79	1.018–7.619	
	p for trend					0.041			0.117
HDL CHOL	≤46	19	501	1			1		
	47–54	15	515	0.77	0.385–1.535		0.78	0.374–1.625	
	55–64	19	506	1.01	0.526–1.937		0.99	0.473–2.065	
	≥65	18	534	0.87	0.452–1.692		1.14	0.529–2.442	
	p for trend					0.824			0.682
LDL CHOL	≤100	10	511	1			1		
	101–119	20	498	2.07	0.959–4.488		2.07	0.909–4.729	
	120–139	17	532	1.61	0.727–3.553		1.44	0.615–3.371	
	≥140	24	515	2.11	0.995–4.489		1.72	0.758–3.892	
	p for trend					0.114			0.348

HbA1c	≤5.3	14	329	1			1		
	5.4–5.5	17	462	0.95	0.457–1.957		0.84	0.390–1.797	
	5.6–5.9	21	688	0.91	0.450–1.835		0.75	0.355–1.579	
	≥6.0	19	577	1.08	0.521–2.235		0.83	0.377–1.811	
	p for trend					0.998			0.453
IOP	≤11.2	6	484	1			1		
	11.3–13.2	19	612	2.35	0.928–5.957		3.34	0.927–12.002	
	13.3–15.2	22	579	2.81	1.124–7.016		5.18	1.487–18.037	
	≥15.3	40	565	4.85	2.027–11.616		7.73	2.235–26.768	
	p for trend					<0.001			<0.001
Central corneal thickness	≤531	17	568	1			1		
	532–556	19	559	1.07	0.550–2.095		0.93	0.421–2.053	
	557–583	23	549	1.26	0.665–2.404		1.05	0.483–2.269	
	≥584	29	556	1.50	0.809–2.776		1.01	0.471–2.180	
	p for trend					0.145			0.847
**Women**									
Age	≤54	107	753	1			1		
	55–62	54	763	0.50	0.354–0.701		0.57	0.358–0.904	
	63–68	34	947	0.25	0.170–0.376		0.35	0.208–0.586	
	≥69	15	884	0.12	0.069–0.207		0.21	0.106–0.405	
	p for trend					<0.001			<0.001
HT	≤147	21	724	1			1		
	148–152	42	935	1.09	0.630–1.879		0.88	0.486–1.594	
	153–156	53	817	1.20	0.697–2.068		1.03	0.571–1.872	
	≥157	94	871	1.52	0.890–2.582		1.35	0.752–2.424	
	p for trend					0.056			0.195
BMI	≤20.3	77	809	1			1		
	20.4–22.3	51	827	0.74	0.509–1.075		0.76	0.481–1.205	
	22.4–24.6	38	846	0.60	0.399–0.901		0.58	0.344–0.977	
	≥24.7	44	847	0.67	0.455–0.993		0.68	0.399–1.156	
	p for trend					0.020			0.120
SBP	≤108	76	768	1			1		
	109–120	49	870	0.77	0.523–1.119		0.74	0.458–1.191	
	121–132	40	812	0.84	0.552–1.263		0.87	0.520–1.457	
	≥133	43	877	1.00	0.657–1.510		1.02	0.605–1.729	
	p for trend					0.411			0.698
TG	≤66	48	701	1			1		
	67–89	43	752	1.01	0.652–1.550		0.85	0.530–1.352	
	90–124	36	759	0.93	0.585–1.460		0.77	0.455–1.288	
	≥125	35	751	0.88	0.558–1.399		0.94	0.547–1.619	
	p for trend					0.548			0.624
HDL CHOL	≤56	30	745	1			1		
	57–65	42	680	1.47	0.905–2.391		1.37	0.810–2.298	
	66–75	44	745	1.32	0.815–2.134		1.25	0.735–2.116	
	≥76	46	793	1.22	0.755–1.961		1.02	0.583–1.785	
	p for trend					0.617			0.801
LDL CHOL	≤107	38	707	1			1		
	108–127	30	752	0.86	0.520–1.407		1.07	0.629–1.830	
	128–149	57	735	1.63	1.057–2.521		2.07	1.273–3.352	
	≥150	37	769	0.95	0.591–1.527		1.27	0.744–2.149	
	p for trend					0.497			0.121
HbA1c	≤5.4	54	722	1			1		

	5.5–5.6	49	715	1.22	0.805–1.838		1.29	0.833–1.993	
	5.7–5.8	25	678	0.79	0.473–1.311		0.85	0.493–1.463	
	≥5.9	34	848	0.91	0.570–1.461		1.03	0.618–1.731	
	p for trend					0.435			0.738
IOP	≤11.6	26	697	1			1		
	11.7–13.6	55	867	1.54	0.953–2.502		2.23	1.226–4.052	
	13.7–15.6	61	806	1.84	1.145–2.967		2.25	1.213–4.157	
	≥15.7	56	840	1.52	0.941–2.468		2.33	1.239–4.367	
	p for trend					0.109			0.023
Central corneal thickness	≤524	54	812	1			1		
	525–550	44	790	0.77	0.510–1.173		0.83	0.508–1.361	
	551–574	42	803	0.72	0.470–1.092		0.70	0.423–1.164	
	≥575	60	799	0.99	0.674–1.463		0.92	0.561–1.523	
	p for trend					0.953			0.730

Non high myopia: SEq >-6D, High myopia: SEq ≤-6D. OR 1: Adjusted by age. OR 2: Adjusted by age, HT, BMI, SBP, TG, HDL CHOL, LDL CHOL, HbA1c, IOP and central corneal thickness. HT = height, BMI = body mass index, SBP = systolic blood pressure, TG = triglyceride, HDL CHOL= high-density lipoprotein cholesterol, LDL CHOL= low-density lipoprotein cholesterol, HbA1c = Hemoglobin A1c, IOP = intraocular pressure.
